# Nutritional Aspects of the Association of Spineless Cactus and Urea with Tifton-85 Hay in Wethers’ Diets

**DOI:** 10.3390/ani15192865

**Published:** 2025-09-30

**Authors:** Robert Emilio Mora-Luna, Ana María Herrera, Michelle Christina Bernardo de Siqueira, Maria Gabriela da Conceição, Juana Catarina Cariri Chagas, Thayane Vitória Monteiro Santos, José Augusto Bastos Afonso da Silva, Francisco Fernando Ramos de Carvalho, Marcelo de Andrade Ferreira

**Affiliations:** 1 Departamento de Ciencias Animales, Facultad de Agronomía y Sistemas Naturales, Pontificia Universidad Católica de Chile, Santiago 7820436, Chile; robert.mora@uc.cl; 2 Facultad de Medicina Veterinaria, Universidad San Sebastián, Santiago 8420524, Chile; 3 Departamento de Zootecnia, Universidade Federal Rural de Pernambuco (UFRPE), Recife 52171-900, Brazil; michelle.siqueira2@gmail.com (M.C.B.d.S.); thayane.monteiro@gmail.com (T.V.M.S.); jose.basilva@ufrpe.br (J.A.B.A.d.S.); francisco.rcarvalho@ufrpe.br (F.F.R.d.C.); marcelo.aferreira@ufrpe.br (M.d.A.F.); 4 Centro de Ciências Agrárias e da Biodiversidade, Universidade Federal do Cariri, Juazeiro do Norte 63133-610, Brazil; maria.conceicao@ufca.edu.br; 5 Faculty of Veterinary Medicine and Animal Science, Department of Applied Animal Science and Welfare, Swedish University of Agricultural Sciences, 907-36 Umeå, Sweden; juana.chagas@slu.se

**Keywords:** digestibility, feeding behaviour, roughage, sheep, semi-arid

## Abstract

**Simple Summary:**

In semi-arid conditions, the spineless cactus is an excellent feed option because of its high water-use efficiency for dry matter production and its rich content of non-fibre carbohydrates. This study assessed the replacement of Tifton-85 hay with increasing levels of spineless cactus plus urea and ammonium sulphate in diets for rumen-fistulated wethers, using a roughage:concentrate ratio of 70:30. Replacing up to 44% of roughage with spineless cactus plus urea and ammonium sulphate improved organic matter and metabolisable energy intakes. Digestibility of dry matter, organic matter, and non-fibre carbohydrates increased linearly with the inclusion of spineless cactus plus urea and ammonium sulphate. It also reduced voluntary water intake and feeding time. On the other hand, spineless cactus inclusion increased water excretion in faeces and the degradation rate of dry matter. A combination of 41% spineless cactus plus urea and ammonium sulphate and 29% Tifton-85 hay in a 70:30 roughage:concentrate diet maximised metabolisable energy intake under these conditions. These findings highlight the potential of spineless cactus as a strategic feed component for small ruminants in water-limited environments.

**Abstract:**

This study evaluated the effects of including 0, 150, 300, 450, and 600 g/kg of dry matter (DM) of spineless cactus (SC; *Nopalea cochenillifera* Salm-Dyck) plus urea and ammonium sulphate (UAS) (9:1), replacing Tifton-85 hay (*Cynodon* spp. cv. Tifton 85), on nutrient intake and digestibility, feeding behaviour, water intake, and rumen dynamics. Five rumen-fistulated and cannulated crossbred wethers were randomly assigned in a 5 × 5 Latin square design. A roughage:concentrate ratio of 70:30 was supplied. Organic matter (OM) and metabolisable energy (ME) intakes showed quadratic responses (*p* < 0.05), with maximum values of 1157 g/day and 14.50 MJ/day estimated at SC+UAS levels of 364 and 410 g/kg DM, respectively. Apparent digestibilities of DM, OM, and non-fibre carbohydrates, as well as water excretion in faeces and degradation rate of DM, increased with SC+UAS inclusion (*p* < 0.05). Indigestible neutral detergent fibre (NDF) intake, feeding and rumination times, voluntary water intake, NDF degradation and passage rates, as well as the indigestible NDF passage rate, decreased with SC+UAS inclusion (*p* < 0.05). In wethers’ diets with a roughage:concentrate ratio of 70:30, a roughage combination of SC+UAS and Tifton-85 hay in a 41:29 ratio is recommended to maximise ME intake.

## 1. Introduction

Semi-arid regions cover 22.6 million km^2^ or 15.2% of the Earth’s land surface [1]. From 1948 to 2008, these regions expanded significantly [2]. Using the dataset for this 60-year period, together with 27 climate model simulations, ref. [3] concluded that drylands will continue to expand throughout the 21st century. They projected that, by the end of this century and under a high greenhouse gas emission scenario, global drylands will be 5.8 million km^2^ (or 10%) larger than in the 1961–1990 climatology. Major expansions of drylands are seen over regions of North America, the northern fringe of Africa, the Mediterranean, southern Africa, coastal regions of Australia, the Middle East and central Asia (e.g., Iraq, Iran, Afghanistan) and South America (especially eastern Brazil, southern Argentina and coastal Chile) [4].

In South America, the Brazilian semi-arid region spans 788,064 km^2^, accounting for 48% of the country’s Northeast Region. This area has long been marked by droughts: in the 16th century alone, five drought periods occurred, totalling 8 years of drought. From then until the 20th century, 54 drought periods were recorded, amounting to 100 years of drought [5]. In the 21st century, 8 years of drought have been documented so far, the most recent being from 2012 to 2016, during which accumulated rainfall fell below 500 mm. This resulted in water stress and reduced water availability for vegetation [6], as well as limiting the supply of feed and water for livestock [7]. Given that small-scale farming and livestock ranching in the region depend on rainfall, these industries are especially vulnerable to future droughts [6]. It is therefore essential to adapt cropping and livestock systems to these environmental conditions [7].

In arid and semi-arid environments, where day and night temperatures can vary drastically, cacti are considered highly adapted to drought conditions [8]. These plants utilise crassulacean acid metabolism in their photosynthetic process, which enhances the assimilation of atmospheric CO_2_ in water-restricted terrestrial habitats. Their water use efficiency ranges from 100 to 150 kg H_2_O per kg of dry matter (DM), which is significantly higher than that of C_3_ plants (700–800 kg H_2_O/kg DM) and C_4_ plants (250–350 kg H_2_O/kg DM) [9].

Spineless cactus (SC) can be utilised in ruminant feeding, and its use dates back to the early decades of the 20th century, when it was provided in combination with sorghum (hay and/or silage), and in some instances even served as the sole feed for dairy cows [10,11]. At that time, its high palatability and dry matter digestibility (62%), as well as its elevated mineral content (18.21%), were particularly noteworthy. SC has been the subject of intensive study in countries such as Mexico [12,13], Morocco [14,15], Tunisia [16,17], and Brazil [18,19,20].

SC is one such plant well adapted to the conditions of the Brazilian semi-arid region. It also demonstrates high palatability for ruminant species (cattle, sheep, and goats) and can be voluntarily consumed in large quantities [21]. SC is a roughage characterised by a high content of non-fibre carbohydrates (NFC) (527–662 g/kg DM) and low levels of DM (96–186 g DM/kg fresh basis), neutral detergent fibre (NDF) (201–268 g/kg DM), and crude protein (CP) (34–58 g/kg DM) [19,22,23,24]. Moreover, its high moisture content is advantageous considering the region’s limited water resources because it reduces the need for additional water supply. However, because of its low NDF and CP content, feeding programmes that include SC must combine it with another roughage source to provide physically effective fibre (PEF) and protein [25], or with non-protein nitrogen sources [21], such as a mixture of urea and ammonium sulphate (UAS) [26]

Tifton-85 hay (*Cynodon* spp. cv. Tifton 85) has high NDF content (770 g/kg DM) [27] and serves as a roughage option and a source of PEF to be combined with SC, as it is one of the commercially produced forages in the Northeast Region of Brazil, typically cultivated in areas with water availability and irrigation [28]. It was therefore hypothesised that there exists a proportion of SC+UAS:Tifton-85 hay that maximises nutrient intake and digestion. The objective of this study was to evaluate the effect of including SC (*Nopalea cochenillifera* Salm-Dyck) + UAS in wethers’ diets based on Tifton-85 hay on nutrient intake and digestibility, feeding behaviour, water intake, and rumen dynamics.

## 2. Materials and Methods

The experiment was conducted at the Animal Science Department of the Federal Rural University of Pernambuco, located in Recife, Pernambuco State, Brazil (08°01′13.4″ S and 34°57′14.9″ W). The climate is classified as tropical hot and humid (Am) according to the Köppen–Geiger system [29]. The site is situated at an altitude of 4 m, with an average annual precipitation of 1804 mm and an average annual temperature of 27.5 °C, ranging from 24 °C to 31 °C.

The Tifton-85 hay was purchased from the local market in Recife, Pernambuco, Brazil, while SC was supplied by the Caruaru Experimental Station of the Agronomic Institute of Pernambuco, located in Caruaru (semi-arid climate), Pernambuco, approximately 130 km from Recife. The SC was harvested every 14 days and transported to Recife for storage *in natura*, as its chemical composition remains unchanged for up to 60 days after harvest [30].

The animals used in the experiment were registered and cared for in accordance with the guidelines and recommendations of the Committee of Ethics on Animal Studies at the Federal Rural University of Pernambuco (Licence No. 069/2016).

### 2.1. Animals, Management, and Experimental Design

Five rumen-fistulated and cannulated crossbred wethers (of no defined breed), averaging 43.8 ± 5.80 kg in body weight (BW) and 14 ± 2.33 months of age, were randomly assigned to five treatments in a 5 × 5 Latin square design. The animals were treated for internal parasites before the start of the experiment and housed in individual pens (0.93 × 1.54 m), each equipped with a feeder and water supply. Diets were offered ad libitum as a total mixed ration twice daily, at 08:00 and 16:00 h. The amount of feed provided was adjusted daily to allow for refusals of approximately 5–10% of the total DM offered. The experiment lasted 110 days, divided into five experimental periods of 22 days each, comprising 14 days for diet adaptation [31] and 8 days for sampling and data collection.

The chemical composition of the dietary ingredients is shown in Table 1.

The experimental treatments are shown in Table 2 and consisted of a control diet with Tifton-85 hay as the sole roughage source, alongside diets in which 20%, 40%, 60%, and 80% of the Tifton-85 hay was replaced by SC plus a mixture of UAS (9:1). These corresponded to dietary DM inclusions of 150, 300, 450, and 600 g SC+UAS/kg DM, respectively. The SC was processed using a crusher (MC-1001N; Laboremus, Campina Grande, Brazil). The concentrate portion of the diet included soybean meal, ground corn, common salt, and a mineral mixture, maintaining a roughage:concentrate ratio of 70:30 (Table 2). Based on the treatments, the roughage fraction (70%) consisted of the following SC+UAS:hay ratios: 0:70, 15:55, 30:40, 45:25, and 60:10, corresponding to 0, 150, 300, 450, and 600 g SC+UAS/kg DM, respectively. All diets were formulated to be isonitrogenous (14% CP) and to meet the nutritional requirements for sheep with an average daily gain of 250 g/day [32].

### 2.2. Data and Sample Collection

Roughage and concentrate were offered, and refusals were weighed daily (Ramuza Scale, Model Ramuzatron 15; Santana de Parnaíba, São Paulo, Brazil) from the 15th to 22nd day of each experimental period to estimate nutrient intake. Samples of feeds and refusals were collected. To estimate the apparent digestibility of nutrients (DM, CP, NDF, and NFC), total faeces collection was conducted from the 17th to 19th day of each period using collection bags adapted to the animals’ bodies. The procedure followed the methodology originally described by [33] for cattle, with size modifications and adjustments appropriate for small ruminants. Faeces were removed from the collection bags every 6 h. During these 3 days, following the morning feeding, total urine collection was also performed. Funnels and hoses were attached to the abdominal area and penis of the animals to collect urine in containers. At the end of each collection period, the total urine volume and weight were recorded, and a 50-mL aliquot was stored at −20 °C [34].

Voluntary water intake was measured daily before the morning feeding from the 17th to 19th day of each experimental period. Five kilograms of water were offered daily at 08:00 h, and after 24 h, refusals were measured and discarded, followed by a fresh supply of clean water. To estimate evaporation losses, two buckets—each containing 5 kg of water—were placed in two empty pens adjacent to those housing the experimental animals. Feeding behaviour was assessed using the scan sampling method [35] on the 15th day of each experimental period. Wethers were observed every 10 min over a 24 h period, beginning immediately after the morning feeding. Each animal’s activity was recorded as either rumination, feeding, or idling.

On the 20th and 22nd days of each experimental period, the rumen evacuation technique was carried out [36]. All rumen contents were manually removed at 12:00 h on the 20th day and at 08:00 h on the 22nd day. Following rumen emptying, the total weight of the digesta was recorded, and the material was filtered through four layers of cheesecloth to separate the solid and liquid phases. Representative samples of both phases were collected and frozen at −20 °C for later analysis of DM, NDF, and indigestible NDF (iNDF) contents. After sampling, the solid and liquid phases were remixed, and the remaining digesta was returned to the rumen. Samples of feeds, refusals, faeces, and ruminal content (solid and liquid phases) were pre-dried in a forced-air oven (Model TE 394-2; Tecnal, Piracicaba, Brazil) at 55 °C until reaching a constant weight, for subsequent chemical analysis.

### 2.3. Chemical Analyses

Samples of feeds, refusals, faeces, and ruminal content were ground using a mill (Model MA 340; Marconi, Piracicaba, Brazil) with 1 and 2 mm sieves (2 mm for iNDF determination) for chemical analysis. According to [37], the samples were analysed for DM by gravimetric estimation at 105 °C (method 930.04), CP (N × 6.25) by the Kjeldahl method (method 984.13), and ash by ignition at 600 °C (method 942.05). Crude fat was determined using the submersion method in hexanes [38]. All these chemical analyses were performed on samples processed with the 1 mm sieve. The water content of urine, as well as the DM content of ruminal samples, was determined by gravimetry at 105 °C.

To determine the iNDF concentration and iNDF intake, feed and refusal samples (processed using a 2 mm sieve) were incubated in the rumen of a bovine for 288 h [39] followed by determination of the NDF concentration. NDF in ingredients, refusals, faeces, and rumen-incubated material was analysed using heat-stable amylase (Termamyl 2X; Novozymes, Copenhagen, Denmark) without sodium sulphite [40]. The NDF was corrected for nitrogenous compounds [41] and expressed as ash-free organic matter (OM) [40]. Acid detergent fibre and lignin in all ingredients were determined according to [42], with the exception that lignin concentration in SC was determined using potassium permanganate oxidation [43].

### 2.4. Calculations

The chemical compositions of the diets were calculated based on the proportions of the ingredients and their respective values. NFC and total carbohydrates (TC) were calculated according to [44] and [45], respectively. OM was calculated as OM = 1000 g/kg DM − g ash/kg DM. Intakes of DM, OM, CP, NDF, and NFC were calculated by subtracting the amounts present in the refusals from the daily amounts offered.

The apparent digestibility of DM and its constituents was calculated as the difference between the amount consumed and the amount excreted in faeces, divided by the amount consumed. Total digestible nutrient (TDN) intake was calculated according to [45] and converted, according to [32], into digestible energy (1 kg TDN = 18.45 MJ of digestible energy), which was then used to estimate metabolisable energy (ME) as: ME = digestible energy × 0.82).

Feeding and rumination efficiencies (based on DM and NDF) were calculated by dividing DM intake (DMI) and NDF intake (NDFI) by the respective times spent feeding and ruminating. Voluntary water intake was determined as the difference between the amount of water offered daily and the amount refused, with adjustments made for evaporative losses measured using control buckets. Preformed water intake was estimated as the difference between *in natura* feed intake and DMI. Metabolic water production was estimated based on digested nutrients, assuming that 40, 50, and 107 g of water are produced per 100 g of oxidised protein, carbohydrate, and fat, respectively [46].

The ruminal contents of DM, NDF, and iNDF were quantified. To determine the flow of particles within the rumen, iNDF was used as an internal marker [47]. The rumen dynamics rates for DM and NDF were estimated following the method described by [36]: intake rate (*Ki* = g·h^−1^ consumed/g rumen pool), passage rate (*Kp* = flow g·h^−1^/g rumen pool), and digestion rate (*Kd* = *Ki* − *Kp*).

### 2.5. Statistical Analysis

Data were analysed using the MIXED procedure of SAS Version 9.4 (SAS Institute, Cary, NC, USA) following a 5 × 5 Latin square design. Orthogonal polynomial contrasts were applied to evaluate linear and quadratic effects. Differences were considered statistically significant at *p* < 0.05. The statistical model was

*Y_ijk_* = *µ* + *T_i_* + *P_j_* + *A_k_* + *E_ijk_*, (1)

where *Y_ijk_* is a dependent variable, *µ* is the mean for all observations, *T_i_* is the fixed effect of diet *i*, *P_j_* is the random effect of period *j*, *A_k_* is the random effect of animal *k*, and *E_ijk_* ~*N*(0, *σ*^2^*_e_*) represents the residual error.

For variables that exhibited a quadratic response, regression analysis was used to fit the curve and obtain the corresponding equation. The maximum SC+UAS inclusion level was determined by setting the first derivative of the quadratic model (y = ax^2^ + bx + c) equal to zero. The estimated inclusion level was then substituted into the equation to calculate the predicted maximum response for the parameter evaluated.

## 3. Results

### 3.1. Nutrient Intake and Apparent Digestibility

The intake of OM and ME showed a quadratic increase (*p* < 0.05) (Table 3), with maximum values of 1157 g/d, and 14.5 MJ/d, respectively, corresponding to SC+UAS inclusion levels of 364 and 410 g/kg DM. Conversely, NDF intake decreased with increasing SC+UAS inclusion (*p* < 0.01), while NFC intake increased. CP intake was not significantly affected (*p* > 0.05). With the exception of CP and NDF (*p* > 0.05), the apparent digestibility of DM, OM, and NFC increased as the inclusion of SC+UAS increased (*p* < 0.01) (Table 3).

### 3.2. Feeding Behaviour

Feeding and rumination times, as well as the feeding and rumination efficiencies for NDF, decreased with increasing inclusion of SC+UAS (*p* < 0.01). By contrast, idle time and the feeding and rumination efficiencies for DM increased (*p* < 0.01) (Table 4).

### 3.3. Water Intake and Excretion

Water excretion in faeces, as well as preformed and total water intakes, increased with increasing SC+UAS inclusion (*p* < 0.01) (Table 5). By contrast, voluntary water intake and the ratio of voluntary water intake to DMI showed a decrease (*p* < 0.01). Metabolic water production exhibited a quadratic response (*p* < 0.05), with a peak of 0.550 L/day observed at 333.3 g SC+UAS/kg DM. Urinary volume and urinary water excretion were not affected by SC+UAS inclusion (*p* > 0.05).

### 3.4. Ruminal Dynamics

Fresh matter in the rumen decreased with increasing SC+UAS inclusion (*p* < 0.05) (Table 6). Similarly, the ruminal pools of DM, NDF, and iNDF, as well as the proportion of DM in the rumen, showed a decline (*p* < 0.05). An increase was observed in the *Ki* and *Kd* of DM (*p* < 0.01) with SC+UAS inclusion, whereas the opposite behaviour was observed in the *Kp* of DM; *Ki*, *Kp*, and *Kd* of NDF; and *Kpi* of iNDF (*p* < 0.05).

## 4. Discussion

### 4.1. Nutrient Intake and Apparent Digestibility

The quadratic effect observed indicates that OM intake increased up to a level of 364 g SC+UAS/kg DM (Table 3), likely due to a reduction in the iNDF content of diets as the inclusion of SC+UAS increased, resulting improvement in diet digestibility. As noted by [48], the intake of indigestible material and rumen fill tend to decline once feed digestibility surpasses 700 g/kg DM—a trend also evident in this study. Given the chemical composition of SC (Table 1) and the experimental diets (Table 2), an increase in NFC intake and a decrease in NDFI were expected in diets with higher levels of SC.

The decrease in OM intake at 364 g SC+UAS/kg DM, may be attributed to metabolic regulation. Diets containing SC have been shown to increase ruminal concentrations of propionic acid [49], which can reduce feed intake because absorbed propionic acid influences satiety [50]. Interestingly, ME intake reached its peak (14.5 MJ/day) at 410 g SC+UAS/kg DM, which is higher than the level at which maximum OM intake occurred (364 g SC+UAS/kg DM), even though OM intake declined beyond that point. The increase in NFC intake and its digestibility (Table 3) likely contributed to a higher TDN intake and, consequently, greater ME intake.

The higher apparent digestibility of DM and OM was likely due to the high effective ruminal DM degradability of SC (711 g/kg DM) [25]. This can be attributed to its low NDF content (148 g/kg DM), high NFC concentration (712 g/kg DM), and low lignin content (10 g/kg DM), all of which contribute to greater digestibility. SC demonstrated a digestibility of 774 g/kg DM [51], in contrast to Tifton-85 hay, which has a higher NDF content (651 g/kg DM), greater lignin content (62 g/kg DM), and lower digestibility (443–555 g/kg DM) [27].

SC has low PEF, as demonstrated by [52] in cattle. In their study, wheat bran (518 g/kg DM of the diet) with an NDF content of 327 g/kg DM was entirely replaced by SC (292 g NDF/kg DM) mixed with UAS, and no change in rumination time was observed. This finding suggests that SC and wheat bran have comparable PEF. Notably, the PEF of wheat bran corresponds to approximately 33% of its NDF content—a much lower proportion than that of forages, which can range from 73 to 98% of NDF content [53], depending on particle size.

In this experiment, despite the lower amount of NDF provided by Tifton-85 hay—considered a source of physically more effective fibre—at the higher levels of SC+UAS inclusion compared with the zero inclusion level (65 vs. 452 g/kg DM) (Table 2), the apparent digestibility of NDF was not affected. This may be attributed to the similarity in potential NDF degradability over 72 h of incubation between SC (789 g/kg DM) [25] and Tifton-85 hay (719 g/kg DM) [54]. In addition, the lower supply of PEF may have been offset by the greater effective degradability of SC’s NDF (degradation rate of 5%/h) compared with that of Tifton-85 hay (514 vs. 418 g/kg DM) [25,54].

### 4.2. Feeding Behaviour

The reduction in feeding time (Table 4) with increasing SC+UAS inclusion can be attributed to the high palatability of SC, which enables animals to consume large quantities more rapidly. Additionally, when SC is processed using a forage machine, its mucilage becomes exposed and adheres to other dietary ingredients, thereby reducing feed selectivity [21].

The low NDF content in diets containing SC may also contribute to reduced feeding time because lower NDF levels are associated with shorter eating durations [55]. In addition, the moisture content of roughage affects consumption rate. On a fresh basis, moist roughages are consumed 6 to 7 times faster than hay, and 2.5 times faster on a DM basis [56]. In this experiment, the high moisture content of SC (777 g/kg fresh basis) increased the overall moisture of the diets—from 78 to 675 g/kg on a fresh basis—as Tifton-85 hay was progressively replaced by SC.

The reduction in rumination time with increasing SC+UAS inclusion is likely due to the decline in dietary NDF content as Tifton-85 hay was replaced by SC (Table 2), resulting in reduced PEF. The influence of NDF intake on rumination time is well documented [24,57,58]. The observed increases in feeding and rumination efficiencies of DM with SC+UAS inclusion can be attributed to higher DMI over a shorter period and reduced rumination time, respectively. Conversely, the decreases in feeding and rumination efficiencies of NDF were a result of the lower NDF content in SC (Table 1), which led to reduced dietary NDF levels and, consequently, lower NDF intake.

### 4.3. Water Intake and Excretion

The increase in preformed water intake corresponded with the rise in dietary moisture content as Tifton-85 hay was progressively replaced by SC. Voluntary water intake (L/day, L/kg DMI, and mL/BW^0.75^) decreased with SC+UAS inclusion, consistent with previous findings [59,60,61]. This underscores the significance of SC as a water source under semi-arid conditions—even when used alone. Indeed, it was observed that withholding water from sheep fed diets containing 300, 500, and 700 g SC/kg DM did not affect their productive performance [62].

The minimum voluntary water intake observed in this experiment was 0.555 L/day at the inclusion level of 600 g SC+UAS/kg DM. According to the literature [61,62], lower voluntary water intake has been reported in sheep fed SC, with values of 0.2 and 0.22 L/day for diets containing 500 and 700 g SC/kg DM, respectively. The higher voluntary water intake recorded in the present study compared with these previous findings may be attributed to the higher DM content of the SC used here (223 g DM/kg fresh matter), which lowered the overall moisture content of the diets (675 g/kg fresh matter at the maximum inclusion level). As a result, animals compensated by increasing voluntary water intake. By contrast, the SC used in the other studies had lower DM content (131–140 g DM/kg fresh matter), which increased the dietary moisture content to between 772 and 810 g/kg fresh matter, thereby reducing the need for voluntary water intake.

The quadratic behaviour observed in metabolic water production reflected a similar quadratic response in total digestible carbohydrate intake (*p* = 0.009), with values of 545, 657, 845, 739, and 772 g for SC+UAS inclusion levels of 0, 150, 300, 450, and 600 g/kg DM, respectively. The maximum intake was estimated at 804 g, corresponding to an inclusion level of 434 g SC+UAS/kg DM. Total digestible carbohydrates accounted for an average of 85.8% of the metabolic water produced.

To estimate water intake (preformed water intake + voluntary water intake) in sheep, [63] proposed the equation water intake = 0.32 × DMI + 1.49, applicable when grass hay with a DM content of 859 g/kg fresh matter is used as feed. In this experiment, for diets containing 0 g SC+UAS/kg DM—closely matching the DM content of the forage used to derive the equation—the estimated water intake was 1.85 L/day. However, as shown in Table 5, the actual water intake observed was higher than predicted, at 2.371 L/day.

The same author proposed an alternative equation for silage-based diets (309 g DM/kg fresh matter: water intake = 3.86 × DMI − 0.99. Applying this equation, water intakes of 3.62 and 3.77 L/day were estimated for diets containing 450 and 600 g SC+UAS/kg DM, respectively. However, the actual values observed in this study were lower—3.099 and 3.392 L/day, respectively (Table 5). This suggests that the animals’ water requirements may already have been met under these conditions. Therefore, when estimating water intake in sheep, special attention must be paid to both environmental conditions and the DM content of the diet. Notably, the original equations were developed under average ambient temperatures of 4.0 °C and 13.2 °C for the silage- and hay-based equations, respectively, whereas the present experiment was conducted at an average temperature of 27.5 °C.

Ref. [61] highlighted the diuretic effect of SC, attributed to its high potassium content. However, in the present study, the inclusion of SC+UAS did not affect either urinary volume or urinary water excretion. This contrasts with findings from other studies [22,59,64], where the inclusion of SC in the diet led to an increase in urinary volume.

The inclusion of SC+UAS leads to increased water excretion in the faeces. When animals are fed SC, preformed water intake rises while voluntary water intake declines. This increase in water intake—unregulated by the animal—may affect water absorption mechanisms in the large intestine [65]. In studies using increasing levels of dehydrated SC in sheep diets, refs. [66,67] have observed higher faecal moisture content. These findings suggest that preformed water intake from SC may independently alter water reabsorption processes in the large intestine when SC is used as a feed component.

### 4.4. Ruminal Dynamics

The reduction in the liquid phase of the rumen may indicate an increased passage rate of this phase, as diets with SC+UAS inclusion resulted in higher total water intake (preformed plus voluntary water). The decrease in the ruminal pool of DM, NDF, and iNDF, as well as in the composition of ruminal DM, aligned with the chemical composition of the diets, as similarly noted by [68]. According to [69], the size of the ruminal NDF pool is the most accurate indicator of rumen fill or capacity because non-fibre DM occupies minimal space in the rumen. Thus, the observed reduction in the NDF pool supports the interpretation that the decline in OM intake beyond the inclusion of 364 g SC+UAS/kg DM was a result of metabolic regulation.

The respective increase and decrease in the *Ki* of DM and *Ki* of NDF reflect the same pattern observed in the feeding efficiencies of DM and NDF, respectively. Although OM intake exhibited a quadratic response (Table 3), a linear decrease was observed in the *Kp* of NDF (Table 6), which was unexpected because increased feed intake is typically associated with a rise in the *Kp* of NDF [70]. This unexpected result may be linked to the reduction in the rumen’s liquid phase. A potentially higher passage rate of the liquid phase could have supported increased OM intake up to its peak, after which intake declined, likely due to metabolic regulation.

The decrease in the *Kp* of DM and NDF may be attributed to the reduction in rumination time (Table 4) because rumination is essential for breaking down large particles, thereby facilitating their passage from the rumen [71]. Ref. [72] found that primary mastication during eating accounted for the breakdown of approximately 25% of large forage particles, while secondary mastication during rumination contributed to a further 50% reduction.

The decrease in the *Kd* of NDF may be linked to a potential decline in ruminal ammonia nitrogen concentrations with the inclusion of SC in the diet [73], as a strong relationship has been reported between ammonia nitrogen levels and fibre digestion (R^2^ = 0.9218) [70]. However, ammonia nitrogen primarily influences the activity of microorganisms adhered to feed particles, which play a greater role in the digestion of cellulose and hemicellulose, rather than free-floating rumen microbes [74]. Thus, even if ruminal ammonia nitrogen concentrations were low, they may still have been adequate to support the activity of adhered microorganisms in degrading other diet components, such as NFC [70]. Furthermore, NFC intake increased with SC+UAS inclusion (Table 3), which helps explain the observed increase in the *Kd* of DM.

The decrease in the *Kp* of NDF may have enhanced NDF digestion in the rumen by extending its residence time, even though a reduction in the *Kd* of NDF was also observed. This can be explained by the fact that, in diets low in PEF, the reduction in the size of fibrous particles relies primarily on microbial activity rather than mechanical breakdown [75]. This mechanism helps account for the similar apparent digestibility of NDF across the diets.

## 5. Conclusions

In wethers’ diets with a roughage-to-concentrate ratio of 70:30, the inclusion of 600 g SC+UAS/kg DM (SC+UAS:hay ratio of 60:10) increases NFC intake, as well as the digestibility of DM, OM and NFC. It also enhances feeding and rumination efficiencies for DM, total water intake and the digestion rate of DM. However, at this inclusion level, OM and ME intakes decrease. Therefore, to optimise nutrient utilisation, a roughage composition consisting of spineless cactus (with urea and ammonium sulphate) and Tifton-85 hay in a 41:29 ratio is recommended to maximise ME intake.

## Figures and Tables

**Table 1:** Chemical composition of ingredients (g/kg DM).

Item	Tifton-85 Hay	Spineless Cactus	Soybean Meal	Ground Corn	Urea	Ammonium Sulphate
DM ^a^	929	223	918	899	991	994
Ash	103	92	74	16	-	-
OM	897	908	926	984	-	-
CP	118	33	518	100	2900	1295
Crude fat	14	15	16	44	-	-
NDF	651	148	97	80	-	-
iNDF	298	80	1.6	4.0	-	-
ADF	366	98	85	30	-	-
Lignin	62	10	4.3	10	-	-
NFC	113	712	295	761	-	-
TC	765	860	392	841	-	-

^a^ g/kg of fresh weight. DM: dry matter; OM: organic matter; CP: crude protein; NDF: neutral detergent fibre corrected for ash and nitrogenous compounds; iNDF: indigestible NDF; ADF: acid detergent fibre; NFC: non-fibrous carbohydrates; TC: total carbohydrates.

**Table 2:** Proportion of ingredients and chemical composition of the experimental diets.

Ingredients (g/kg DM)	Inclusion of SC+UAS (g/kg DM)				
	0	150	300	450	600
Tifton-85 hay	693	549	399	250	99
Spineless cactus	0	147	293	437	583
Ground corn	208	204	203	204	205
Soybean meal	84	81	82	82	82
Urea+Ammonium sulphate ^a^	0	4	8	12	16
Common salt	5	5	5	5	5
Mineral mix ^b^	10	10	10	10	10
Chemical composition (g/kg DM)					
DM ^c^	922	629	478	387	325
OM	919	921	923	925	927
CP	146	143	140	141	140
Crude fat	20	20	20	21	21
NDF	476	404	327	252	175
iNDF	208	176	143	111	77
ADF	267	229	188	148	107
NFC	277	361	449	534	621
TC	753	757	762	765	769
NDF by Tifton-85 hay	452	358	260	163	65

^a^ 9 parts urea and 1 part ammonium sulphate. ^b^ Assurance levels provided by the manufacturer: (g/kg) 120 Ca, 87 P, 147 Na, 18 S; (mg/kg) 590 Cu, 40 Co, 20 Cr, 1800 Fe, 80 I, 1300 Mn, 15 Se, 3800 Zn, 10 Mo, and 870 F (maximum). ^c^ g/kg of fresh weight. DM: dry matter; OM: organic matter; CP: crude protein; NDF: neutral detergent fibre corrected for ash and nitrogenous compounds; iNDF: indigestible neutral detergent fibre; ADF: acid detergent fibre; NFC: non-fibrous carbohydrates; TC: total carbohydrates.

**Table 3:** Nutrient intake and apparent digestibility in sheep fed spineless cactus plus urea and ammonium sulphate.

Item	Inclusion of SC+UAS (g/kg DM)					SEM	*p*-Value	
	0	150	300	450	600		L	Q
Intake (g/d)								
OM	1003	1061	1240	1061	1104	70.5	0.104	0.014 ^a^
CP	167	172	195	165	175	11.5	0.609	0.065
NDF	504	441	400	255	200	31.9	<0.001	0.227
NFC	328	458	672	684	789	34.3	<0.001	0.896
ME (MJ/d)	11.3	12.4	16.1	14.1	15.5	0.94	<0.001	0.025 ^b^
Digestibility (g/kg DM)								
DM	673	710	781	776	804	17.5	<0.001	0.168
OM	682	733	794	793	822	16.9	<0.001	0.122
CP	768	770	791	784	806	13.5	0.055	0.832
NDF	585	586	639	532	540	38.2	0.239	0.313
NFC	798	880	899	912	918	15.7	<0.001	0.202

OM: organic matter; CP: crude protein; NDF: neutral detergent fibre corrected for ash and nitrogenous compounds; NFC: non-fibre carbohydrates; ME: metabolisable energy; DM: dry matter; SEM: standard error of the mean; L: linear; Q: quadratic. ^a^ OM = −0.0012(SC+UAS)^2^ + 0.8737(SC+UAS) + 997.97. ^b^ ME = −0.00002(SC+UAS)^2^ + 0.0164(SC+UAS) + 11.131.

**Table 4:** Feeding behaviour in sheep fed spineless cactus plus urea and ammonium sulphate.

Item	Inclusion of SC+UAS (g/kg DM)					SEM	*p*-Value	
	0	150	300	450	600		L	Q
Feeding time (min/d)	246	216	242	170	184	16.8	<0.001	0.789
Rumination time (min/d)	562	482	488	398	328	42.1	<0.001	0.482
Idle time (min/d)	632	742	710	872	928	44.4	<0.001	0.472
Feeding efficiency								
DM (g/h)	274	333	349	436	411	35.2	<0.001	0.187
NDF (g/h)	123	124	100	94.3	66.6	10.9	<0.001	0.094
Rumination efficiency								
DM (g/h)	119	147	175	187	232	13.9	<0.001	0.679
NDF (g/h)	53.5	55.2	50.8	40.8	37.0	4.00	<0.001	0.106

DM: dry matter; NDF: neutral detergent fibre; SEM: standard error of the mean; L: linear; Q: quadratic.

**Table 5:** Water intake and excretion in sheep fed spineless cactus plus urea and ammonium sulphate.

Item (L/d)	Inclusion of SC+UAS (g/kg DM)					SEM	*p*-Value	
	0	150	300	450	600		L	Q
Preformed water intake (A)	0.072	0.838	1.723	2.140	2.837	0.17	<0.001	0.234
Voluntary water intake (B)	2.298	1.886	1.497	0.960	0.555	0.19	<0.001	0.852
Water intake (A+B)	2.369	2.724	3.220	3.099	3.392	0.21	0.002	0.317
Metabolic water (C)	0.470	0.529	0.617	0.536	0.543	0.04	0.048	0.006 ^a^
Total water intake (A+B+C)	2.840	3.252	3.838	3.636	3.934	0.22	0.001	0.195
Urinary volume	1.523	1.717	1.512	1.459	1.606	0.20	0.871	0.933
Water excretion								
Urine	1.494	1.762	1.479	1.436	1.581	0.20	0.778	0.992
Faeces	0.509	0.616	0.708	0.717	0.717	0.06	0.007	0.143
Voluntary water intake								
L/kg DMI	2.223	1.596	1.085	0.801	0.458	0.17	<0.001	0.196
mL/BW^0.75^	130.9	105.9	83.66	52.33	32.26	11.8	<0.001	0.987

DMI: dry matter intake; BW: body weight; SEM: standard error of the mean; L: linear; Q: quadratic. ^a^ Metabolic water = −0.0000009(SC+UAS)^2^ + 0.0006(SC+UAS) + 0.4694.

**Table 6:** Ruminal pool and rates of ingestion, passage, and digestion of some nutrients.

Item	Inclusion of SC+UAS (g/kg DM)					SEM	*p*-Value	
	0	150	300	450	600		L	Q
Fresh matter (g)								
Solid	1428	1220	1202	1122	929.1	108	0.004	0.935
Liquid	3258	3100	2890	2926	2554	201	0.001	0.690
Total	4686	4320	4092	4048	3483	238	<0.001	0.691
Ruminal pool (g)								
DM	578.2	513.8	515.2	493.8	431.0	41.4	0.023	0.893
NDF	363.8	300.8	289.4	271.2	215.8	27.9	0.002	0.932
iNDF	211.6	174.4	154.2	141.0	111.4	20.9	0.002	0.752
Composition of ruminal DM (g/kg)								
NDF	625.6	586.0	558.3	545.4	499.8	14.1	<0.001	0.958
iNDF	360.0	337.5	297.0	281.6	257.4	21.2	<0.001	0.725
DM (h^−1^)								
*Ki*	0.0806	0.0952	0.1128	0.1048	0.1198	0.0066	0.001	0.295
*Kp*	0.0354	0.0326	0.0330	0.0206	0.0198	0.0035	<0.001	0.414
*Kd*	0.0452	0.0628	0.0796	0.0846	0.1002	0.0047	<0.001	0.395
NDF (h^−1^)								
*Ki*	0.0591	0.0610	0.0595	0.0414	0.0384	0.0048	0.001	0.133
*Kp*	0.0226	0.0217	0.0222	0.0143	0.0144	0.0025	0.004	0.424
*Kd*	0.0366	0.0393	0.0372	0.0271	0.0239	0.0029	0.001	0.075
iNDF (h^−1^)								
*Kpi*	0.0458	0.0459	0.0488	0.0338	0.0347	0.0054	0.039	0.373

iNDF: indigestible NDF; *Ki*: ingestion rate; *Kp*: passage rate; *Kd*: digestion rate; *Kpi*: passage rate of iNDF; SEM: standard error of the mean; L: linear; Q: quadratic.

## Data Availability

The raw data supporting the conclusions of this article will be made available by the authors on request.
